# Editorial: Sustainable digital economy, entrepreneurship, and blockchain technology role in industrial-organizational psychology

**DOI:** 10.3389/fpsyg.2022.974415

**Published:** 2022-07-15

**Authors:** Muddassar Sarfraz, Larisa Ivascu, Muhammad Ibrahim Abdullah

**Affiliations:** ^1^School of Management, Zhejiang Shuren University, Hangzhou, China; ^2^Faculty of Management in Production and Transportation, Politehnica University of Timisoara, Timişoara, Romania; ^3^Department of Management Sciences, COMSATS University Islamabad, Lahore, Pakistan

**Keywords:** digital economy, entrepreneurship, blockchain technology, industrial-organizational psychology, sustainability

With the advancement in information technology, today's digital economy offers numerous services influencing the global sectors. A sustainable digital economy, entrepreneurship, and blockchain have inevitably compelled the entire business infrastructure to alter the digital market dynamics, influencing industrial-organizational psychology. Today, digitalization has provided ample opportunities to promote a sustainable mindset. The emergence of information technology has shaped the business dynamics, thereby digitally empowering organizations to experience the features of sustainable innovation. These momentous developments in the digital era have put forward new ideas, fostering the characteristics of today's enterprises to encourage conventional businesses to embrace novel transformations.

With the advent of globalization, the digital economy has extensively expanded worldwide markets, emphasizing the importance of incorporating digital ecosystems. The recent technological surge has driven the market toward sustainable digital development. Indeed, in the wake of this digitalization, the debate on a sustainable digital economy has gained prominence, causing the concepts of entrepreneurship and blockchain technology to gain researchers' attention. Today's digitization has encouraged the key players to adapt to the changing modern market conditions that focus on innovation and sustainability. This fundamental shift has prompted the world's businesses to shift from traditional commerce to online forums.

A call for papers on this topic was made by the Journal of frontier in psychology. Energized by the emerging significance of this Research Topic, numerous contributors heeded the call. In a Research Topic, researchers contributed to a discussion on *Sustainable Digital Economy, Entrepreneurship, and Blockchain Technology Role in Industrial-Organizational Psychology*. The current study examines the 23 relevant articles from that issue that fundamentally fulfill the research objective. These papers include articles from different disciplinary sectors (e.g., industrial, agricultural, and educational). The submissions were collected from diverse geographical places such as China, Pakistan, France, the United States, and Qatar, and offer a detailed view of this Research Topic. As such, this paper uncovers a significant understanding of the sustainable digital economy, entrepreneurship, and blockchain technology influencing industrial-organizational psychology.

Notably, this Research Topic illuminates the progress made on this subject during recent years. The prevalent scholarly perspective encourages global economies to embrace innovative technology, thus enhancing industrial psychological welfare. Indeed, the range of articles presented under this theme open new avenues for future researchers. This distinct theme embedded in this Editorial article provides a brief overview of the gathered contributions supporting the call. We hope that all the papers work as an inspiration, thus providing directions to future researchers.

In recent decades, the role of technology has flourished due to the effect of social media on our lives. Social media's impact on marketing is a key area of study for today's researchers. It has fundamentally transformed conventional business practices to shape customers' purchasing behavior. In recent years, e-business and social media networking has profoundly altered the market competition, strongly encouraging consumers to accept the offered product *via* social media. Marketing through social media has also gained attention in the world of entrepreneurship. This innovative technology (i.e., social media) has enabled consumers to modify their purchasing behavior toward achieving a sustainable digital economy. In explaining this notion, Dong et al. showed how today's digital marketing techniques have positively influenced consumers' online purchases to establish a sustainable digital approach.

In line with the past findings, there has also been recent progress in service marketing. Service is the most fundamental aspect that influences consumers' buying decisions. With the development in the digital world, service marketing has extended beyond the physical space to the virtual marketplace in various forms. The introduction of digital platforms for online activities has emerged as a useful tool for applying business practices. The novel blockchain technology has brought about changes in the business dynamics, thereby promoting online buying. This technological support has potentially encouraged businesses to enhance traditional marketing practices with innovative technological infrastructures. This new model radically strengthens online buying by establishing global payment methods. Undoubtedly, this value proposition has evolved businesses to take advantage of the novel digital platforms to increase and enhance business interactions (Zutshi et al., 2021). However, despite its increasing significance, online shopping has made it difficult for consumers to evaluate the quality of their purchases. Such unclear standards have elevated the need to make service marketing workable for consumers, thus helping them make wise decisions. Therefore, Wang Y. et al. suggest that organizations should revive their service marketing strategies to reflect consumers' online purchase experience.

Over the years, blockchain technology has emerged as an innovative technological tool, drastically transforming firms' conventional business models into potential innovative systems. In this regard, today, the internet has brought numerous advantages for organizations, thus ensuring the proper functioning of business models. In the wake of this digitalization, blockchain has optimized business value chains (Milashovska et al., 2021), substantially fostering changes in firms' business activities (Purusottama et al., 2022). This growing popularity of information technology has encouraged individuals to use digital tools for online purchases. Liu states that this technological advancement has made people switch from physical buying to online buying. In entrepreneurship, online shopping has attracted large markets, thus leading this mode of purchase to become today's norm. However, despite the increasing significance of social media marketing, online stores have faced considerable competition, causing individuals to encounter uncertain scenarios during online shopping. Guo et al. explain that leveraging the e-shopping experience requires management to improve the quality of their services to improve their customers' purchases due to loyalty.

Altogether, the economy has gained the benefits of these technologies and is currently transitioning from conventional forms to digital forums. The impact of blockchain technology may have intensified business activities, accelerating consumers' consumption patterns (Ertz and Boily, 2019). Overall, blockchain technology boosts participants' trust in business transparency, thereby fulfilling the needs of individual shoppers. The online platforms operating with the help of blockchain technology accelerate business engagement among platform users, profoundly centralizing individuals' trust in the business offering.

Undoubtedly, blockchain technology has rapidly conquered the world. Today, its increasing significance has led organizations to focus on creating value for their stakeholders. Also, its impact has encouraged firms to capitalize on the digital transformation, by promoting operational efficiency and innovation. This change toward a digital standard has greatly disturbed the conventional activities of global organizations, especially ones involving customer relationships. Digital marketing has driven businesses' transformational characteristics to enhance individuals' experiences (Varma et al., 2021).

This development in e-commerce has caused countries across the globe to experience sustainable advancement in business functions. Recently, digitalization has modified the online medium to contribute to sustainable growth. It has encouraged global economies to capitalize on firms' marketing activities with novel developments. Indeed, today, digital marketing has become a global phenomenon driving businesses toward sustainable digital development. In supporting this notion, Pei states that firms' digital marketing is a key aspect in their achievement of sustainable development. In particular, creating a reliable and trustworthy distribution system has become almost compulsory for today's organizations. In this modern world, the global economic system depends upon integrating novel digital tools that create, store, and accelerate firms' business models. These developing blockchain applications encourage organizations to promote their products, substantially leading to a sustainable economy (Beck et al., 2017).

In the wake of digitalization, technology has played a vital role in the sustainable development of many countries. For example, Wang and Zhao suggest that this technological boost requires corporations to understand today's novel innovations to gain enduring competitiveness. Undoubtedly, there is a global consensus that digitalization leads to a sustainable digital economy. Among the different ideas presented on the digitalized economy, sustainable development is a dominant concept confronting the modern world. A sustainable digital economy enables organizations to scale up their objectives, thus leading to an increase in firms' information technology growth. In this regard, Xianbin and Qiong state that organizations should embrace novel reforms to build a sustainable digital economy.

Achieving enduring sustainability is the biggest challenge facing today's entrepreneurs. Accordingly, in recent years, entrepreneurship has gained researchers' attention. The entrepreneurial philosophy has encouraged stakeholders to alter their decisions regarding their corporate social responsibility (CSR) practices. From the viewpoint of entrepreneurship, Wei et al. state that CSR and innovation have broadly made the stakeholders appreciate and value the effect of financial performance on firms' CSR activities. Moreover, in this era of ITC innovation, CSR policies have compelled organizations to reinforce, normalize, and eliminate social, economic, and ecological disparities, thus contributing to stakeholders' welfare (Bapuji et al., 2020). As a result, governments and companies have embraced CSR activities to resolve market uncertainties (e.g., environmental and social concerns). However, despite the increasing role of CSR, global governments have raised concerns about firms' CSR activities. In this context, e-government has emerged as a phenomenon, enhancing the effectiveness of service delivery. It has made the government responsible for transparent societal activities. In explaining this notion, Avotra et al. reveal a negative influence of e-government on firms' corporate social responsibility, thereby emphasizing the need for stakeholders to adopt effective e-governmental policies.

In particular, the global trend has encouraged countries to contribute to sustainable growth, with specific projects, such as those of CPEC, being a common way of achieving sustainability. Across the journey of China-Pakistan Economic Corridor (CPEC), several innovative projects have started under this initiative. Numerous countries have responded to the increasing CPEC benefits, thus making global institutions realize their significant influence on the world's environment. Indeed, today, CPEC has made densely populated countries fulfill their responsibility toward environmental protection. Accordingly, Xiaolong et al. suggest that CPEC projects should ensure the world's ecological conditions to boost economic welfare. However, besides the CPEC growth, industrialization has greatly affected the world's climate and individuals' health. In this regard, Gherheş et al. suggest that waste material should be reduced, by ensuring firms' eco-friendly actions to ensure sustainable development. Further, the continuous improvement in individuals' living standards demands enhanced medical services and, in turn, improved health ensures individuals' career growth. Ge et al. state that individuals' career growth largely depends on individuals' motivation, health, and self-efficacy.

Notably, in the twenty-first century, rapid changes have pushed companies to ensure a healthy environment in which employees can flourish. In this regard, emotional intelligence has played a fundamental role in enhancing employees' quality of living. The emotions relating to the Big Five Personality Model improve employees' living standards. In illustrating this notion, Dan et al. explain that the partial effect of the Big Five models of personality on emotional intelligence influences employees' entrepreneurial behavior. Moreover, Linfang et al. also state that personality traits (i.e., extraversion, agreeableness, openness, conscientiousness, and neuroticism) enhance women's entrepreneurial intentions, thus accelerating their self-leadership.

In entrepreneurship, the progressing trend in digital technologies has profoundly changed the business dynamics, significantly influencing business infrastructures. Today's IT solutions have allowed the technological paradigm to enhance entrepreneurial activities, thereby empowering modern firms to achieve entrepreneurial success. The rigorous diffusion of technology has encouraged a new breed of entrepreneurs who launch novel ventures supporting business growth. As such,  reveals that firms' technological knowledge and entrepreneurial orientation fundamentally drive their succeArdeleanss. Due to these factors, value modules have become a global trend in fostering business success. Modularity supplements firms' knowledge foundations, thereby accelerating their performance. In understanding this notion, Wang J. et al. suggest that value modularity influences firms' innovation performance and business growth.

Over the years, several technological phenomena have influenced conventional commercial exchanges, reconfiguring the concept of collaborative economies to experience the benefits of blockchain technologies. This direct intensification has underpinned the robust digital infrastructures to increase the use of new technologies by redefining business processes. Concerning the connected economy, the smart grid of technological advancements (i.e., blockchain) has illuminated innovative payment methods to lead companies toward sustainable economic welfare (Ertz and Boily, 2020). Blockchain, introduced as a management technology, has taken over the world's economic welfare activities. Its decentralized system has profoundly updated organizations' prior processes, thus bringing sustainable benefits. Its potential in various sectors has enhanced the digital economic system, potentially ensuring a country's sustainable development. The development of information technology has sped up the digital economy to an unprecedented extent. Firms' technological freedom has allowed employees to express themselves in democratic management practices, substantially enhancing their organization's economic value. Based on this statement, Jiang et al. reveal that, in the digital economy, employees' direct and indirect involvement drastically alters firms' management practices, thus bringing positive financial results.

In this regard, the origin of financial inclusion has considerably gained prominence in the global sectors. The thematic concept of financial inclusion enhances businesses' access to consummating financial resources. Therefore, there is an immense need to embrace financial inclusion across worldwide industries, thus achieving sustainable growth. Financial inclusion helps to eradicate poverty, thus assisting businesses to contribute to achievement of the Millennium Development Goals (Valencia et al., 2021). In exploring this phenomenon, Liu et al. state that individual households should use financial inclusion to overcome the challenges hindering individuals' economic freedom. Another study conducted by Han and Gu states that digital financial inclusion enhances the innovative performance of high-tech companies, thereby achieving sustainable digital growth.

In recent years, the COVID-19 pandemic has caused unprecedented vulnerabilities influencing firms' sustainable growth in the educational sector. COVID-19's high rate of infectivity has drastically disturbed the educational system, due to the closure of many worldwide institutions. However, in combatting the increasing challenges of COVID-19, today's educators have widely advocated the inclusion of information technology as part of the learning system. Fundamentally, today's novel digital implementations have drastically impacted students' learning. The e-learning system has considerably filled the gap by facilitating learning over the web. Digital learning has seen significant uptake in recent years, encouraging institutions to shift their traditional learning system to digital forums. A study initiated by Zhang states that the pandemic has changed the whole scenario, leading the global education system to go online, and the effectiveness of pandemic learning has developed as a result.

In the education sector, mentors play a significant role in influencing a student's life, as individuals need direction to improve their vision. In this regard, entrepreneurial mentorship has emerged as a successful tool for providing training to the individual. Mentorship is a global phenomenon that enhances individuals' competencies by constantly developing their capacities. The mentor's assistance stimulates the individual's knowledge-sharing behavior, thus ensuring growth in their career. For example, Zhao indicates that mentorship plays a significant role in fostering an individual's knowledge-sharing behavior. In digital entrepreneurship, the mentorship upgrades the knowledge system to combat the emerging entrepreneurship challenges. Another study shows that the sustainable approach to entrepreneurial mentorship alters farmers' education and behavior in the agricultural sector. Hu et al. state that farmers' education, training, and motivation accelerate their behaviors to influence the firms' entrepreneurial success.

Moreover, during the pandemic crises, the global media played a major role in reporting the lethal nature of the outbreak, prompting fear in individuals but also spreading important awareness regarding the situation. Arguably, providing news with the aid of social media is the prime responsibility of today's media firms. However, during the pandemic, many groups used such platforms to spread false information. In supporting this statement, Wang K. et al. revealed that social media played a key role in spreading news during the COVID-19 pandemic, driving fear among individuals.

In conclusion, this study summarized 23 papers, with each paper contributing significantly to upgrading the past knowledge on the proposed theme. This study gives an overview of the literature by outlining the studies from different domains. All of the papers analyzed add value to the previous literature. We expect these papers to guide the researchers, professionals, organizations, and policymakers. This special issue has undoubtedly inspired numerous writers to share their perspectives in the Journal of the Frontier of Psychology. In particular, we hope that all the articles expand the understanding of the intended subject. Indeed, we expect this initiative to serve as a beneficial model for future researchers. Fundamentally, it will enable them to address the questions that have remained unclear regarding sustainable digital economy, entrepreneurship, and blockchain technology's role in different industries. Altogether, we thank all the researchers for taking this idea forward with their multi-disciplinary research.

## Author contributions

All authors listed have made a substantial, direct, and intellectual contribution to the work and approved it for publication.

## Conflict of interest

The authors declare that the research was conducted in the absence of any commercial or financial relationships that could be construed as a potential conflict of interest.

## Publisher's note

All claims expressed in this article are solely those of the authors and do not necessarily represent those of their affiliated organizations, or those of the publisher, the editors and the reviewers. Any product that may be evaluated in this article, or claim that may be made by its manufacturer, is not guaranteed or endorsed by the publisher.
